# Alterations in the Composition of Intestinal DNA Virome in Patients With COVID-19

**DOI:** 10.3389/fcimb.2021.790422

**Published:** 2021-11-24

**Authors:** Zhen-Hua Lu, Hao-Wei Zhou, Wei-Kang Wu, Ting Fu, Min Yan, Zhen He, Shi-Wei Sun, Zhao-Hua Ji, Zhong-jun Shao

**Affiliations:** ^1^ Department of Epidemiology, Ministry of Education Key Lab of Hazard Assessment and Control in Special Operational Environment, School of Public Health, Air Force Medical University, Xi’an, China; ^2^ School of Public Health, Baotou Medical College, Baotou, China; ^3^ School of Public Health, Gansu University of Chinese Medicine, Lanzhou, China

**Keywords:** COVID-19, gut virome, bacteriome, bacteriophage, virus-bacteria linkages

## Abstract

Patients with Coronavirus Disease 2019 (COVID-19), due to severe acute respiratory syndrome coronavirus 2 (SARS-CoV-2) infection mainly present with respiratory issues and related symptoms, in addition to significantly affected digestive system, especially the intestinal tract. While several studies have shown changes in the intestinal flora of patients with COVID-19, not much information is available on the gut virome of such patients. In this study, we used the viromescan software on the latest gut virome database to analyze the intestinal DNA virome composition of 15 patients with COVID-19 and investigated the characteristic alternations, particularly of the intestinal DNA virome to further explore the influence of COVID-19 on the human gut. The DNA viruses in the gut of patients with COVID-19 were mainly crAss-like phages (35.48%), *Myoviridae* (20.91%), and *Siphoviridae* (20.43%) family of viruses. Compared with healthy controls, the gut virome composition of patients with COVID-19 changed significantly, especially the crAss-like phages family, from the first time of hospital admission. A potential correlation is also indicated between the change in virome and bacteriome (like *Tectiviridae* and *Bacteroidaceae*). The abundance of the viral and bacterial population was also analyzed through continuous sample collection from the gut of patients hospitalized due to COVID-19. The gut virome is indeed affected by the SARS-CoV-2 infection, and along with gut bacteriome, it may play an important role in the disease progression of COVID-19. These conclusions would be helpful in understanding the gut-related response and contribute to the treatment and prevention strategies of COVID-19.

## Introduction

With the rapid and wide transmission of severe acute respiratory syndrome coronavirus 2 (SARS-CoV-2) worldwide, Coronavirus Disease 2019 (COVID-19) has become a pandemic. Until July 2021, there have been 181 million confirmed cases of COVID-19, including 3.9 million fatalities globally; the number of infections and deaths is still increasing rapidly and substantially. SARS-CoV-2 can activate innate and adaptive immune responses of the host and result in acute inflammatory responses (Yan et al., 2020), which may lead to local and systemic tissue damage. In fact, the SARS-CoV-2-related symptoms occur not only in the respiratory but also in the gastrointestinal tract (Chen et al., 2020; Huang et al., 2020).

The intestinal microbiota is the largest and the most complex microecosystem in humans, and its composition and functional homeostasis are essential for the maintenance of normal immune function and defense against infection (Kelly et al., 2005; Tappenden and Deutsch, 2007; Qin et al., 2010; Belkaid and Hand Timothy, 2014; Moeller et al., 2016; Schmidt et al., 2018). Phages, eukaryotic viruses, and plant-derived viruses interact with symbiotic bacteria and the intestinal barrier to promote important functions necessary for intestinal health (Reyes et al., 2012; Li et al., 2019; Shkoporov and Hill, 2019). The linkage between virome and bacteriome composition was recently demonstrated (Draper et al., 2018; Moreno-Gallego et al., 2019). The alterations of the gut virome are found to be specific in some gastrointestinal and systemic diseases, such as inflammatory bowel disease (Norman Jason et al., 2015; Zuo et al., 2019), AIDS (Monaco et al., 2016), diabetes (Ma et al., 2018), and malnutrition (Reyes et al., 2015). To date, most of the research on intestinal microbes has focused on the study of bacteria, while the intestinal virome composition and its impact on human health and disease have not been examined utterly. This may be attributed to the fact that bacteria and archaea account for nearly 94% of the total DNA of the gut microbial biomass. Besides, most bioinformatic methods depend on the available database, while up to 86–99% of viral reads remain unknown as the “viral dark matter” (Virgin Herbert, 2014; Roux et al., 2015; Aggarwala et al., 2017) because of the enormous size and diversity of global viral populations.

With the rapid development of high throughput sequencing technology, especially the metagenomics method, it has become possible to further study the host intestinal virome that is not possibly investigated by culture-based methods. As a result, a large amount of human gut metagenomes have been mined in studies conducted recently to provide new insights into the viral diversity of the human gut microbiome. Studies on the gut microbiota of patients with COVID-19 have been ongoing since the last year. However, the relationship between SARS-CoV-2 infection and associated changes in intestinal microbes (including both virome and bacteriome) has not been thoroughly examined. While a few studies have performed metagenomic sequencing on the patient’s intestinal samples to analyze the characteristic of intestinal bacteria, not much attention has been paid to the data generated on intestinal virome through metagenomic sequencing.

In this study, we collected the open accessed fecal metagenome data of hospitalized COVID-19 patients, used bioinformatics tools to analyze the information on intestinal viromes, and attempted to describe the alterations in intestinal virome composition during hospitalization. We also assessed the effects of antibiotic use and disease severity on the composition of intestinal virome and found the potential correlation between virome and bacteriome changes in these patients. Our findings indicate that SARS-CoV-2 infection leads to alterations in the gut virome of COVID-19 patients, which will contribute to the understanding of the COVID-19 pathogenesis and treatment.

## Materials and Methods

### Data Description

The metagenomic data used in this study were obtained from a gut microbiota study of patients with COVID-19 (Zuo et al., 2020), available publicly at the National Center for Biotechnology Information Sequence Read Archive (BioProject accession number PRJNA624223). As described in the original study (Zuo et al., 2020), 15 patients with COVID-19 and 6 pneumonia controls (hospitalized with community-acquired pneumonia) were chosen from hospitalized patients in Hong Kong, China. Additionally, 15 individuals with no past medical history or history of antibiotic intake in the past three months and those tested negative for SARS-CoV-2 were selected as healthy controls (Zuo et al., 2020). The severity of COVID-19 infection in these patients was classified into four groups, based on symptoms, namely (1), mild (five fecal samples from one patient, no radiographic evidence of pneumonia) (2), moderate (32 fecal samples from nine patients, pneumonia accompanied by respiratory tract symptoms) (3), severe (seven fecal samples from three patients, respiratory rate ≥30/min, oxygen saturation ≤93% when breathing, or PaO_2_/FiO_2_ ≤300 mm Hg), or (4) critical (eight fecal samples from two patients, respiratory failure requiring mechanical ventilation, shock, or organ failure requiring intensive care). The stool samples were collected, extracted and sequenced with same methods. In this study, we obtained the quality-controlled and host-removed metagenomic data, along with original information of 15 patients, 6 pneumonia controls and 15 healthy controls (**Table S1**).

### Taxonomic Classification and Abundance Profiling of Gut DNA Virome and Bacteriome

The virome from raw metagenomic reads of first-time sample after hospitalization was taxonomically characterized using Viromescan software after efficiently denoising samples from reads of other microorganisms (Rampelli et al., 2016). Then, a customized database was established to detect the common DNA viruses and phages in the human gut. The reference sequences of common gut DNA viruses were derived from the official reference database of viromescan software. This customized database also contained 142,809 non-redundant and high-quality gut phage genomes (Camarillo-Guerrero et al., 2021). The bacteria were taxonomically classified using kraken2 (Wood and Salzberg, 2014) software. Then the relative abundance was calculated in each sample using Bracken (Lu et al., 2017) software(2.5.0). For each sample, the composition distribution of virome families was plotted using the ggplot2 package in R (version 4.0.2). Group comparisons between sequences from COVID-19 samples and those of healthy controls were performed using the statistical analysis of taxonomic and functional profiles (STAMP) software (http://kiwi.cs.dal.ca/Software/STAMP, version 2.1.3). Dynamic changes in differential viral relative abundance in fecal samples of patients with COVID-19 during hospitalization were normalized to log10 and the visualizations were performed with R package ggplot2.

### Co-Occurrence Network Analysis

Based on relative abundances, eight virus families and the top 40 bacterial families were analyzed to illustrate the association between intestinal virome and bacteriome of patients with COVID-19. The association between the intestinal virome and bacteriome was examined using the Procrustes function in R vegan package (Oksanen et al., 2019), and their correlations were calculated using the Spearman method in the ggClusterNet package (https://github.com/taowenmicro/ggClusterNet); then, an association network graph was generated using igraph packages (Csardi and Nepusz, 2006) (v1.2.5) in R. Significant associations were visualized with Gephi (Bastian et al., 2009) (version 0.9.2), wherein, nodes represent different families of virome and bacteriome, and the edges represent their positive and negative correlations.

### Statistical Analysis

Alpha and beta diversity analyses were performed with the vegan package in the R language (4.0.2). Briefly, the Shannon and Simpson indexes were calculated to represent the alpha diversity of the gut virome. A Mann-Whitney Test was used to compare alpha diversities between two groups. Principal coordinates (PCoA) analysis and constrained PCoA analysis based on Bray-Curtis dissimilarities were performed using permutational multivariate analysis of variance (PERMANOVA) in vegan packages. Welch’s t-test was used for group comparisons analysis in STAMP. Procrustes analysis for both virome and bacteriome was performed based on the Bray-Curtis distances of eigenvalues. The M^2^ value was re-computed 999 times and the p-value was calculated based on the proportion of M^2^ values that were equal to or lower than the actual M^2^ value. Co-occurrence network analysis was employed with p < 0.05 and spearman correlation value > 0.3.

## Results

### Diversity and Composition of the Microbial Community

**Figure 1:**
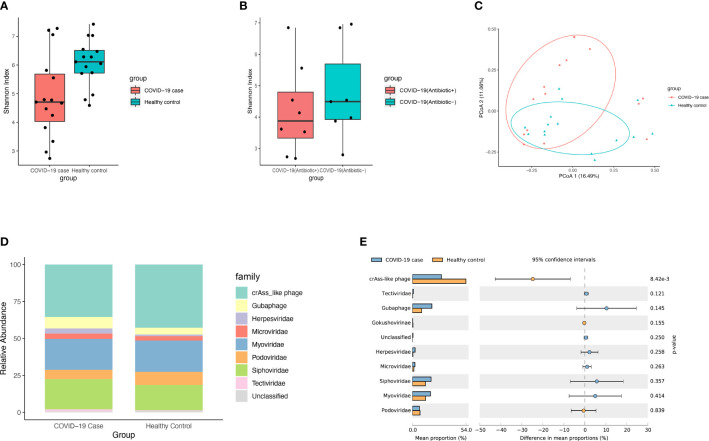
Viral diversity and composition of microbial community. **(A)** Boxplot showed viral alpha diversity between COVID-19 patients and healthy controls based on the Shannon index. The dot points represent the index of each of samples. **(B)** Boxplot showed viral alpha diversity between COVID-19 patients with antibiotic treatment(Antibiotic+) and those without antibiotic treatment(Antibiotic-) based on the Shannon. **(C)** PCoA analysis based on Bray-Curtis distance between COVID-19 patients and healthy controls in the relative abundance of virome. The dot points represent the distance of each of samples from the two most explainable dimensions. **(D)** Viral community structural composition and distribution on family level. The Stack bar diagram represent the percent of taxonomical composition in two groups. **(E)** The extended error barplot shows the abundances of different viral abundances in the two groups of samples. The middle shows the abundances of different species within the 95% confidence intervals. The value on the far right is p-value.

The DNA virome of patients with COVID-19 (n = 15) from the first-time sample of stool collected after hospitalization was compared with that of healthy controls (n = 15). Microbial alpha diversity analyses based on the Shannon (P = 0.016, Mann-Whitney Test, **Figure 1A**) index showed a significant decrease in the gut DNA virome composition in patients with COVID-19. While the microbial alpha diversity showed no significant change between COVID-19 patients with antibiotic treatment (Antibiotic+) and those without antibiotic treatment(Antibiotic-) based on the Shannon index (P > 0.05, Mann-Whitney Test, **Figure 1B**). Further, beta diversity analyses based on Bray-Curtis distance showed a significant difference (R^2^ = 0.071, P= 0.016, PERMANOVA, **Figure 1C**) of microbial diversity between patients with COVID-19 patients and healthy controls. PERMANOVA test showed that the infection of SARS-CoV-2 significantly impacted the composition of gut DNA virome (R^2^ = 0.078, P = 0.036, PERMANOVA, **Figure S1**). While the effect size of other factors such as pneumonia, gender and age were not significant.

For the profiling of virome in patient samples, gut eukaryotic DNA viruses and gut phages reference database were annotated using viromescan. The fecal DNA virome of patients with COVID-19 comprised mainly of crAss-like phages (35.48%), *Myoviridae* (20.91%), *Siphoviridae* (20.43%), Guaphage (7.74%), *Podoviridae* (6.29%), *Microviridae* (3.58%), *Herpesviridae* (3.51%) and *Tectiviridae* (1.29%), and some unclassified viruses (**Figure 1D** and **Table S2**). There was a significant decrease in the abundance of crAss-like phages in the gut of these patients. Interestingly, newly-found viruses like crAss-like phages and Guaphage were largely detected in these samples, indicating the existence of a considerable number of viruses that have not been cultured, whose taxonomic classification needs to be investigated. On the other hand, the top 10 bacteria families detected in these samples were *Bacteroidaceae* (24.38%), *Tannerellaceae* (11.46%), *Lachnospiraceae* (11.15%), *Enterobacteriaceae* (10.04%), *Streptococcaceae* (6.94%), *Ruminococcaceae* (6.05%), *Bifidobacteriaceae* (4.47%), *Rikenellaceae* (4.08%), *Akkermansiaceae* (3.88%), *Actinomycetaceae* (3.12%), and *Eggerthellaceae* (1.81%).

Differential analysis of viral abundance showed a significant decrease in the abundance of CrAss-like phages in patients with COVID-19 (**Figure 1E**, P < 0.05). PCoA analysis of virome abundance based on Bray-Curtis distance of all collected fecal samples during hospitalization showed a significant difference among patients with COVID-19 using antibiotics, those who stopped using antibiotics, and those who did not use antibiotics at all (P = 0.001, PERMANOVA, **Figure S2A**). Likewise, PCoA analysis also identified a significant difference in symptoms and their severities in patients with COVID-19 (P < 0.001, PERMANOVA, **Figure S2B**).

### Co-Occurrence Network Analysis Between Gut DNA Virome and Bacteriome in Patients With COVID-19

**Figure 2:**
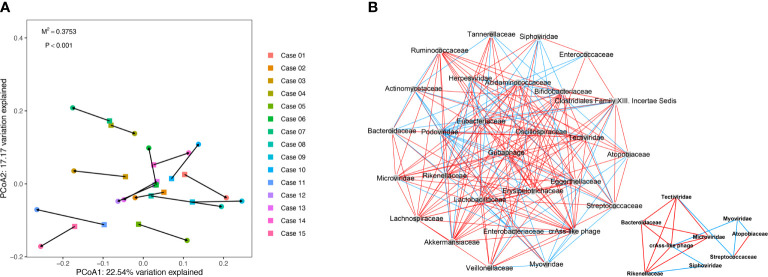
Procrustes analysis and Co-occurrence network analysis between virome and bacteriome in COVID-19. **(A)** Procrustes analysis of the correlation between viral and bacterial communities of COVID-19 patients. **(B)** Co-occurrence network analysis between virome and bacteriome. Each nodes represents virus and bacteria on family levels. Edges represented positive associations (red) and negative associations (green) between virus and bacteria. The cutoff of the Spearman correlation and p-value were set at 0.3 and 0.05, respectively.

To further explore the correlation between gut virome and bacteriome, the gut viral abundance of COVID-19 patients (n = 15, sampled just at the time of hospitalization) were subjected to Procrustes analysis and co-occurrence network analysis with Spearman correlation. The Procrustes analysis, which transforms two distance matrices from corresponding samples to compare distributions, exhibited a strong correlation of 0.652 between viral and bacterial communities in the gut of patients with COVID-19 (Procrustes sum of squares = 0.375, P < 0.001, **Figure 2A**).

We observed a significant correlation between viral and bacterial communities in the gut of patients with COVID-19 (**Figure 2B** and **Table S3**). According to the virus-bacteria co-occurrence network analysis, linkage relationships were indicated between *Tectiviridae* and *Bacteroidaceae* (Spearman r = 0.921, p < 0.05), *Microviridae* and *Bacteroidaceae* (Spearman r = 0.768, p < 0.05), crAss-like phage and *Rikenellaceae* (Spearman r = 0.671, p < 0.05), *Siphoviridae* and *Rikenellaceae* (Spearman r = -0.682, p < 0.05), *Myoviridae* and *Atopobiaceae* (Spearman r = -0.682, p < 0.05), and *Myoviridae* and *Streptococcaceae* (Spearman r = -0.661, p < 0.05). These virus-bacteria linkage relationships, especially the first three linkages, verified the result in the next part of result.

### Alteration in Gut DNA Virome and Bacteriome During Hospitalization

**Figure 3:**
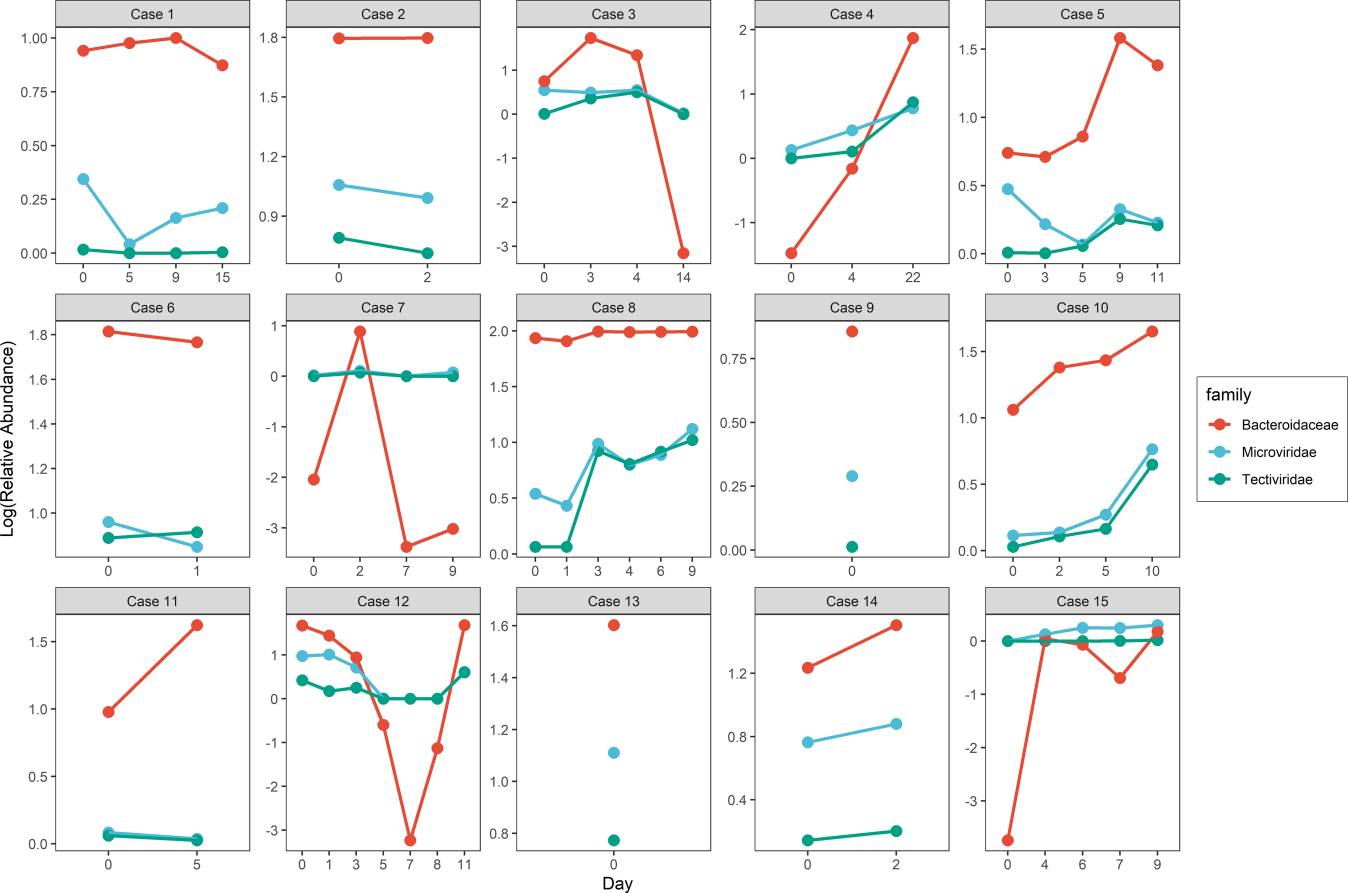
Alteration of viral and bacterial relative abundance in similar trend along hospitalization within COVID-19 patients.

To investigate the change in gut virome composition of these patients with COVID-19, we traced alterations in virome during hospitalization and found an inconsistent tendency of alteration in viral relative abundance over time. A similar phenomenon was also observed in the bacteriome composition of these patients. Noteworthy, the tendency of variation in relative abundance in some families of virus and bacteria was were highly individual-specific. For example, although the range of relative abundance of *Tectiviridae* and *Microviridae* (Spearman r = 0.85, p < 0.001) as well as *Tectiviridae* and *Bacteroidaceae* families (Spearman r = 0.89, p < 0.001) changed with little differences, the trend was similar (**Figure 3** and **Table S4**).

We then traced the data from the original study (Zuo et al., 2020) on fecal SARS-CoV2 viral load and found that the magnitude of the gut virome variation was related to that of the fecal SARS-CoV2 viral load. In most patients with COVID-19, alterations in the gut virome composition gradually leveled off after five days of fecal SARS-CoV2 viral load clearance.

## Discussion

The number of confirmed COVID-19 cases and related deaths has imposed a tremendous burden on the health and economy of the entire international community. There is sufficient evidence that COVID-19 patients endure a fierce immune response such as a cytokine storm (Soy et al., 2020; Ye et al., 2020). SARS-CoV-2 can evade the host immune surveillance mechanism (Shang et al., 2020; Zheng et al., 2020; Thomson et al., 2021), thus, posing challenges in the development of medications and vaccines. On the other hand, the composition and functional homeostasis of the human intestinal microbiota are essential for the maintenance and regulation of normal immune function and resistance to infection (Kelly et al., 2005; Tappenden and Deutsch, 2007). However, until now, studies have only focused on the gut bacteriome rather than gut virome (including abundant phages and viruses infecting eukaryotic cells).

In this study, we characterized the gut DNA virome of patients with COVID-19 by using the viromescan software designed for metagenomics viral community profiling. Particularly, we employed the newest available gut phage database to annotate the taxonomy of gut virome of infected patients, which might help to comprehensively understand the virome community of SARS-CoV-2-infected patients. Although the viromescan software is optimized for eukaryotic viruses, it was still encouraged by author to perform the analysis on phages with database of interest and customize viromescan to work with.

Microbial diversity analysis revealed a significant decrease in diversity of the gut DNA virome in COVID-19 patients compared to healthy controls, which in accordance with similar recent studies (Cao et al., 2021; Zuo et al., 2021). A low diversity of gut microbiota in patients with COVID-19 compared to that in healthy individuals has also been reported earlier (Gu et al., 2020; Zuo et al., 2020; Chen et al., 2021; Vestad et al., 2021). These evidences above supporting the hypothesis that the infection of SARS-CoV-2 impacts the microbial composition and immunity of the host. The impaired angiotensin-converting enzyme 2 (ACE2) expression or function as a result of SARS-CoV-2 infection might contribute to dysbiosis of the intestinal microflora (Hashimoto et al., 2012; Perlot and Penninger, 2013). In fact, the downregulation of ACE2 could reduce the intestinal absorption of tryptophan and the secretion of antimicrobial peptides, which promote pathogen survival and gut dysbiosis (Moal and Servin, 2006; Zhao et al., 2018). In SARS patients, ACE2 expression is downregulated during infection (Kuba et al., 2005). On the other hand, considering that the gut DNA virome was found to mainly comprise bacteriophages, the decreased richness in gut virome of patients with COVID-19 could be interpreted as an adverse scarcity of bacterial hosts (Mukhopadhya et al., 2019).

Other studies on the gut virome of patients with COVID-19 have shown the presence of *Herelleviridae*, *Virgaviridae*, crAss-like phage, *Inoviridae*, *Microviridae*, *Myoviridae*, *Podoviridae*, and *Siphoviridae* family of viruses (Cao et al., 2021). Besides, several phages and eukaryote-associated viruses were profiling in the gut virome of COVID-19 patients in another study (Zuo et al., 2021).

In this study, the gut virome was classified into crAss-like phages, Guaphage, and other viral families including *Myoviridae*, *Siphoviridae Podoviridae*, *Microviridae*, *Herelleviridae*, *Herpesviridae*, and *Tectiviridae*. The current result of gut virome classification revealed the dominance of gut phages, while only a minor proportion was represented by the eukaryote-associated viruses. Bacteriophages comprise over 90% of the human gut virome. Moreno-Gallego et al. found that the gut microbiome is mainly driven by gut phages but not the eukaryotic-associated viruses which are generally disease- or diet-associated (Moreno-Gallego et al., 2019). It was also reported that none of the eukaryotic virus was central in terms of network structure in the gut virome of patients with COVID-19.

Recent advances in viral metagenomics have enabled the rapid discovery of new viruses. CrAss-like phages, the most abundant human-associated virus, have been found in a high proportion which account for about half of human gut viromes (Dutilh et al., 2014; Yutin et al., 2018; Koonin and Yutin, 2020). CrAss-like phages are associated with the phylum *Bacteroidetes* which dominate the human gut microbiome (Koonin and Yutin, 2020). We observed a significant decrease in the relative abundance of crAss-like phages in the COVID-19 patient samples in this study. As the major component of gut virome, a decrease in its relative abundance may indicate the alterations in its bacterial host like *Bacteroidetes*, as mentioned above. Particularly, Guaphage (the gut *Bacteroidales* phage), which was first reported in 2021, was also annotated in the gut virome of patients in this study. The Gubaphage clade is another highly prevalent phage in the human gut (Camarillo-Guerrero et al., 2021), and further culturing and mechanistic studies are needed to improve the understanding of its role in the human gut microbiota.

Antibiotics are commonly used in the initial treatment of SARS-CoV2 infection, which may lead to gut dysbiosis in patients. Our study shows that antibiotics significantly affect human gut virome, consistent with the results of several previous studies (Cao et al., 2021; Yutin et al., 2018). As this is assumed to be a direct response of pathogen burden during the infection (Schneider and Ayres, 2008), we analyzed disease severity to verify its correlation with the composition of gut virome. Concurrent with previous studies, we found a significant difference among symptom severities of COVID-19 patients. Specifically, the relative abundance of nine DNA virus species—in which 7 of them were bacteriophage—in feces correlated negatively with COVID-19 severity. The microbial signature of disease severity such as the depletion of butyrate-producing bacterial groups and the enrichment of opportunistic pathogens was also reported in these studies. These findings suggest the key roles of gut microbiota in the pathophysiology of COVID-19.

Procrustes analysis revealed a correlation between the composition of viral and bacterial communities in patients with COVID-19. Likewise, the co-occurrence network analysis revealed several specific significant linkages between gut virus and bacteria in these patients. This was supported by the findings in mice that accompanying virome shifts are largely localized in mouse bacteriophages and are associated tightly with changes in the bacteriome (Cao et al., 2021).

We found that the alteration in relative abundance in the gut virome was hardly consistent in these COVID-19 patients, while the change in the relative abundance of *Tectiviridae* and *Microviridae* in their gut was more similar to that of the *Bacteroidaceae* family during hospitalization. This phenomenon indicates that the alterations in gut virome might be holistically integrated with gut bacteriome during the progress of immune response against SARS-CoV2 infection. For instance, a mechanism called “lysogenic conversion” could improve the fitness of bacteria when the integrated phage DNA modifies these bacteria. Besides, gut virome, especially the bacteriophages not only directly affect the bacterial populations, but also have an indirect effect on the colonization of their bacterial hosts in the cells (Van Belleghem et al., 2018). We hypothesize that this could be the cause of the similar trend in shifts in the relative abundance of gut bacteria and viruses.

Our study had several major limitations. First, considering that the metagenomic data in this study were DNA reads of human gut microbes, the taxonomic classification in this study was focused mainly on the DNA species of common gut viruses and gut bacteriophages. Most of the gut bacteriophages have been generated and analyzed from large-scale and worldwide-distributed human gut metagenomes, and to date, they are the most comprehensive and complete collection of human gut phage genomes and have been complemented by other published gut phage databases. They can be critical in profiling the gut DNA virome data obtained in this study. Besides, a major difficulty encountered in the analysis of this database was that we could only annotate at the family level because most of the gut bacteriophages have not been cultured yet. Second, as exploratory research, we could not verify a clear cause or consequence effect between gut virome alteration and disease. One of the possible ways for confirmation is by functional validation in animal studies. Third, in the original study, the use of empirical antiviral therapy was reported, including lopinavir-ritonavir (87%), ribavirin (47%), and interferon beta-1b (7%), but not with much individual information. We could only analyze the effect of antibiotic treatments on gut virome during hospitalization, due to the difficulty in more in-depth investigation because of the lack of details in antiviral therapy.

Thus, in future research, it should be important to control these confounding effects while analyzing the gut virome of patients with COVID-19. In addition, considering the limited sample size in the original study, the result of PCoA analysis based on different symptom severities of COVID-19 should be verified in further research with a more reasonable sample collection strategy. In conclusion, this study analyzed the characteristic alternations in intestinal DNA virome composition of patients with COVID-19 to further explore the influence of COVID-19 infection on the human gut. As a result, we found that the gut virome of patients with COVID-19 was significantly altered compared to that of healthy people. Clinical information like disease severity and utilization of antibiotics was analyzed to evaluate their potential influence on the gut virome composition, and several potential indications of correlation between changes in gut virome and flora in COVID-19 patients were observed.

## Conclusion

This study is a preliminary exploration of the alteration in gut DNA virome of individuals during the COVID-19 disease course and the relationship between their gut virome and bacteriome. Noteworthy, this is the first study to examine the gut DNA virome of patients with COVID-19. Given the paucity of research in this area, we believe this study will provide directions for a better and more comprehensive understanding of the disease and potential therapeutic strategies.

## Data Availability Statement

The original contributions presented in the study are included in the article/**Supplementary Material**. Further inquiries can be directed to the corresponding authors.

## Author Contributions

Z-jS and Z-HJ developed the study concept and theory. Z-HL, W-KW, and S-HS performed the bioinformatics analysis. Z-HL, Z-HJ, H-WZ, and W-KW participated manuscript development. TF, MY, and ZH helped to access the public data. All authors participated in the interpretation and presentation of results and have read and approved the final manuscript.

## Funding

This work was supported by the National Natural Science Foundation of China (81803289, 81773488), the Natural Science Foundation of Shaanxi Province (2020JM-329), the Military Medicine Innovation Fund (18CXZ011), and China Special Grant for the Prevention and Control of Infection Diseases (2017ZX10105011). The funding agencies had no role in the study design, data collection and analysis, or preparation of the manuscript.

## Conflict of Interest

The authors declare that the research was conducted in the absence of any commercial or financial relationships that could be construed as a potential conflict of interest.

## Publisher’s Note

All claims expressed in this article are solely those of the authors and do not necessarily represent those of their affiliated organizations, or those of the publisher, the editors and the reviewers. Any product that may be evaluated in this article, or claim that may be made by its manufacturer, is not guaranteed or endorsed by the publisher.
