# Supplementary figures and images for: Alterations in the Composition of Intestinal DNA Virome in Patients With COVID-19
Source: Front Cell Infect Microbiol. 2021 Nov 24;11:790422. doi: 10.3389/fcimb.2021.790422 (PMC8653907; doi:10.3389/fcimb.2021.790422)

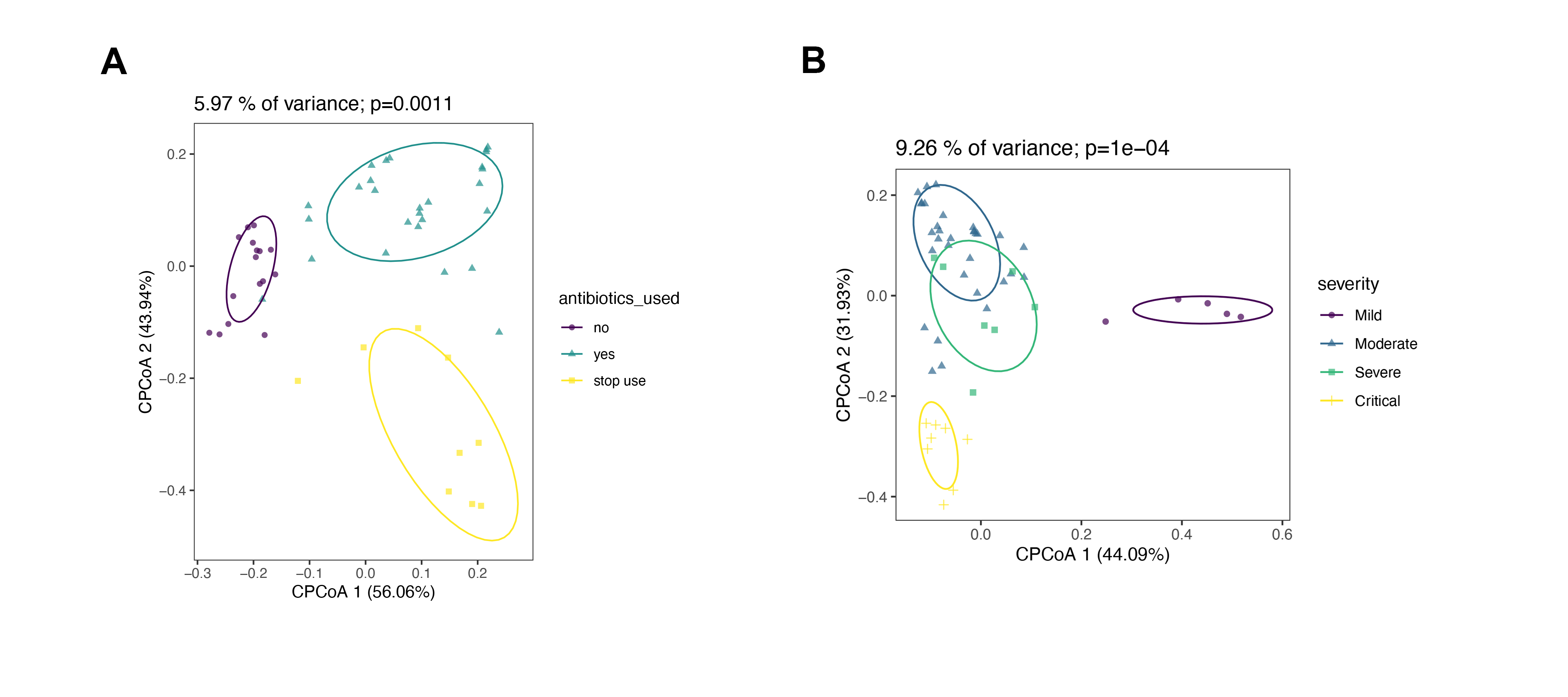

Supplement: Supplementary file 1 [file Image_1.tif]

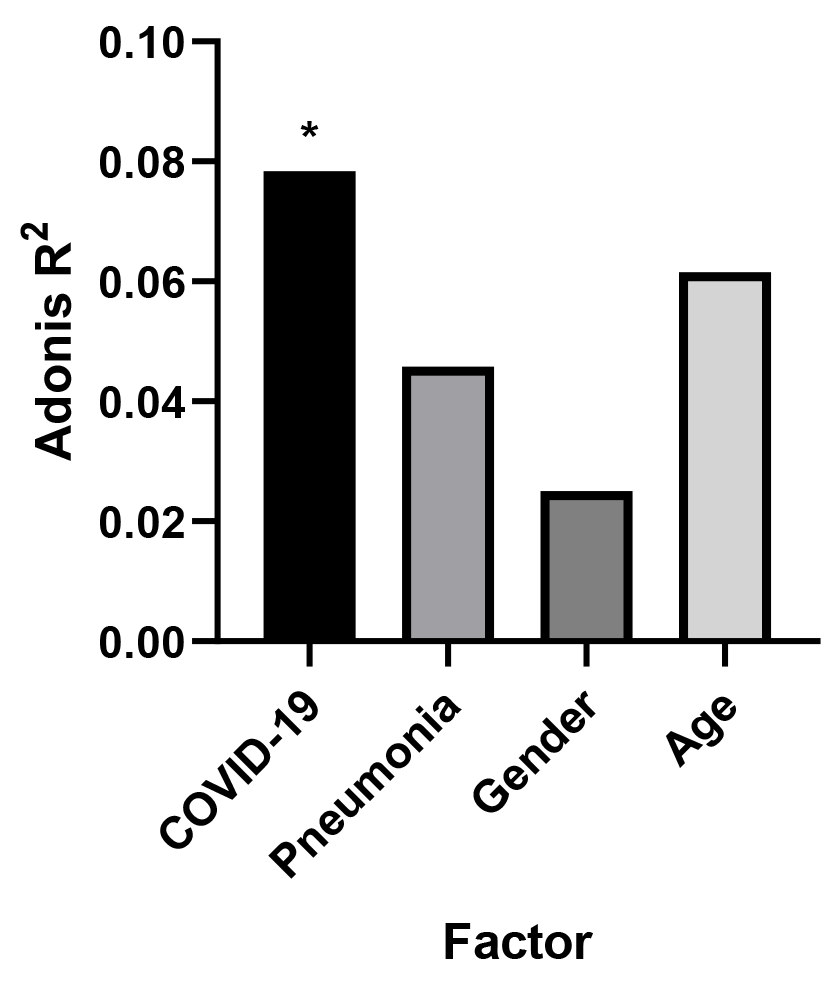

Supplement: Supplementary file 2 [file Image_2.tif]
